# How the innovative advertising language style for nutrition products affects consumers' purchase intentions

**DOI:** 10.3389/fnut.2025.1576478

**Published:** 2025-07-03

**Authors:** Geng Lu, Jun Cao, Yunyun Wei, Dajun Yang, Fuqiang Tan

**Affiliations:** ^1^Digital Industry Research Center, Huainan Normal University, Huainan, Anhui, China; ^2^North Sichuan Medical College, Sichuan Primary Health Service Development Research Center, Key Laboratory of Disease Surveillance and Digital Health Governance, School of Management, Nanchong, Sichuan, China

**Keywords:** advertising language style, purchase intention, information processing fluency, information credibility, nutritional products

## Abstract

**Objective:**

In the competitive market for nutrition products, advertising strategies play a pivotal role in shaping consumer behavior. This study investigated how advertising language styles (disruptive vs. sustaining) influence consumer purchase intentions for nutrition products, examining information processing fluency as a mediator and information credibility as a moderator.

**Methods:**

A multi-group experiment was conducted with 852 participants, recruited via a professional survey platform, in a controlled simulated environment. Data on participants' gender, exposure to advertising language styles (disruptive vs. sustaining), purchase intentions, information processing fluency, and information credibility (high vs. low) were collected using an online questionnaire. PROCESS models (Model 4 for mediation, Model 1 for moderation) and ANOVA (for main effects) were used for statistical analysis.

**Results:**

Disruptive advertising language, compared to sustaining styles, significantly enhanced consumers' attention, interest in product innovations, and purchase intentions. Information processing fluency mediated the positive relationship between advertising language style and purchase intention, highlighting the importance of clarity and ease of understanding.

**Conclusions:**

Disruptive advertising language, fluent information processing, and high information credibility are key factors in enhancing consumers' purchase intentions for nutrition products.

## 1 Introduction

Advertising is becoming increasingly important as an integral part of modern marketing (1). In addition to the basic function of directly conveying product information and attracting consumers' attention, the language style of advertisements also plays a crucial role in shaping brand image (2), and arousing consumers' emotional resonance (3). Nutritional companies have long been exploring how to stand out through innovative advertising that captures and retains consumers' attention (4). In the process, different stylistic styles have been experimented with and employed, aimed at creating a deep connection with the target consumer group and promoting sustainable purchasing behavior (5). New trends in the innovative advertising language style for nutrition productions are emerging, as consumers become more health-conscious and are pursuing technological innovations. In addition, nutrition product advertisements are no longer only emphasizing on the efficacy and ingredients of the products, but also focusing more on emotional expressions and storytelling, to touch consumers' hearts, stimulate their purchase intentions and increase their loyalty (6). Despite the fact that changes in market trends and consumer demands have driven continuous innovations in the language styles of nutrition product advertisements, there is one central question that has still not been properly addressed: are these stylistic changes really able to affect consumers' purchase intentions? In other words, are the subtle and creative stylistic elements in advertisements capable of stimulating consumers' long-term interest and purchasing behavior toward nutrition products? However, research on this issue is still relatively limited and requires further in-depth exploration and validation.

Numerous academic studies have examined the language style of advertisements. These studies have focused on how the advertising language shapes brand image (7), conveys product information (8), and stimulates consumers' interest in purchasing (9). However, most of the current research focuses on how advertising language style directly affects consumers' immediate responses, including the memorability (10), attractiveness (11) of advertisements. There is a lack of research on how the advertising language style affects consumers' purchase intention in the long run, namely, whether consumers will continue to pay attention to and purchase nutrition products of a certain brand, because of advertising language style. Specifically, it has been shown (12) that linguistic features (e.g., phonemes) are effective in affecting consumer perceptions of products (13). On the contrary, if the product advertising language is too bland and lacks novelty, it may be difficult to leave a deep impression in the minds of consumers, which affects their sustained attention and purchasing behavior.

There is still a limited understanding of how innovative advertising language style for nutrition products can subtly influence consumers' psychology as well as their purchase intentions. To fill this gap, we draw on the conceptual framework of language style, which emphasizes that language style in advertisements not only conveys product information, but also profoundly shapes consumers' cognitive and affective responses (14). According to this framework, the language style of innovative advertising for nutrition products, including the application of terminology and the introduction of innovative vocabulary, will give specific symbolic meanings to the advertisement, which in turn resonates with consumers and influence their psychological processes (15). It could be assumed that advertisements with a technology-rich language style may allow consumers to associate the product with positive attributes, such as “high technology” and “health security.” Further, where advertisements adopt a warm, life-affirming style (16), consumers may be more likely to relate to them emotionally, which in turn may increase their brand loyalty and ongoing purchase intentions.

In exploring the impact of innovative advertisements for nutrition products, we focused on its language style, analyzing whether they contain symbolic meanings that can touch consumers' hearts and thus influence their purchase intentions. Drawing on language style theory, we attempt to consider advertising language as a symbolic information carrier (17, 18), which deepens our understanding to the effectiveness of innovative advertising for nutrition product, particularly how to affect consumers' psychological processes through a well-designed language style. The innovative advertising language style for nutrition products can also be analyzed in terms of several dimensions, including professionalism and innovativeness, which shape the overall image of nutrition product advertisements. Based on the above analysis, we assume that the language style of innovative advertisements for nutritional products conveys rich symbolic meanings to consumers, and that when the innovative advertising language style for nutrition products matches their psychological expectations, it in turn promotes the formation of their purchase intentions.

Therefore, this study aimed to provide insights into how, why and when the innovative advertising language style for nutrition products can influence consumers' purchase intentions. Unlike most studies on advertising effectiveness from the external audience perception (19), we adopt the perspective of consumers' information reception of advertising language style for nutrition products and focus on analyzing its subtle impact on consumers' internal psychology and behavioral intentions. Based on language style theory, we constructed a conceptual model to explain how innovative advertising for nutrition products with different language styles (disruptive vs. sustaining) affects the formation mechanism of consumers' purchase intention. Through a series of controlled experiments, we systematically manipulated the advertising language styles and collected data on consumers' immediate feedback and subsequent purchase behavior, to reveal the causal relationship between advertising language styles and consumers' psychological responses. In order to deeply understand the mechanism of advertising language, we also explored the moderating role of information credibility presented in advertisements, so as to provide more comprehensive and precise guidance for advertising strategy-making.

This study has made several important theoretical contributions. First, we formulated a generalized theoretical framework elucidating the effects of language styles (disruptive and sustaining) on nutrition product purchase intentions in innovative advertising. Defining their fundamental dimensions, we show their universal applicability across diverse markets, transcending general impacts and resonating with target consumers. Second, we deepened insights into advertising language styles' mechanisms, uncovering information fluency as a pivotal mediator that bridges language style and purchase intention, fostering smooth information delivery and trust in advertised nutrition products. Third, we uncovered information credibility as a moderator in the language style-purchase intention relationship, enhancing language style impact while encouraging critical evaluation, guiding refined advertising strategies and differentiated marketing.

## 2 Theoretical framework and research hypotheses

In order to achieve the goal of the study, the literature review was accordingly divided into four parts: (1) Language style theory; (2) the mediating role of information processing fluency; and (3) the moderating role of information credibility.

### 2.1 Language style theory

Language style theory refers to a systematic study of the different features formed in language use (20). It involves language expression, rhetorical techniques, stylistic features, aiming to reveal the formation mechanism and characteristics of different language styles, as well as its relationship with factors such as communicative purpose and context (21).

Existing research has found that the impact of language style on consumer product perceptions is multidimensional and complex. On the one hand, language style is directly related to brand image and product positioning. The language style adopted by a brand often reflects the target audience, value proposition, and market positioning of its products (22). For example, high-end brands usually employ a formal, professional language style that conveys quality, professionalism, and credibility, which enhances consumers' recognition of product quality (23). On the other hand, language style affects consumers' emotional resonance. A friendly, humorous, or narrative language style can bring the brand closer to the consumer and trigger emotional resonance, which in turn enhances consumers' interest in the product and their positive feelings (24). This emotional connection helps consumers to develop positive attitudes toward the product, which could be transformed into purchasing behavior. In addition, language style affects how consumers understand and remember product information. Simple, clear, and easy-to-understand language styles help consumers quickly capture the main features and benefits of a certain product and reduce the difficulty of information processing (25). Whereas, overly complex or obscure language styles may confuse consumers or lead to misunderstandings, which may in turn affect their overall evaluation of the product.

This study assumed that, on the one hand, disruptive innovative advertising for nutrition products usually adopts a radical, novel and creative language style, which can quickly capture consumers' attention and stimulate their curiosity and interest. In other words, when unique vocabulary, innovative expressions and compelling sentences are used in advertising, consumers tend to be deeply attracted, thus develop a strong interest in the product. This interest could be transformed into a more positive attitude toward the product and inspire the purchase intentions, as consumers feel the forward-looking, innovative and dynamic nature of the nutrition product. On the other hand, sustaining innovative advertising for nutrition products prefers a language style that is gentle, stable and close to the consumer. The sustaining language style focuses on creating an emotional connection with consumers and enhancing their trust, by conveying the reliability and approachability of a certain brand. When stable and consistent vocabulary and expressions are used in advertising, consumers could feel the credibility of the brand, which leads to the purchase intentions. In addition, sustaining innovative advertising also focuses on details and in-depth exploration of consumers' needs to increase their purchase intentions, by meeting their expectations.

To sum up, disruptive and sustaining language styles of innovative advertising for nutrition products have their own characteristics, and both are capable of positively affecting consumers' purchase intentions. Therefore, the following hypothesis has been proposed.

H1: Disruptive (vs. sustaining) innovative advertising for nutrition products can better inspire consumers' purchase intentions.

### 2.2 The mediating role of information processing fluency

Information processing fluency refers to the perceived difficulty individuals experience when processing information, reflecting the smoothness of the cognitive process (26). In marketing communication, the stylistic features of advertising language—encompassing lexical choices (e.g., vocabulary complexity, concreteness), syntactic structures (e.g., sentence length, grammatical simplicity), and even typographical presentation—can directly affect consumers' information processing fluency, which is distinct from the substantive content of the information itself. A language style characterized by clarity, conciseness, and appropriate vocabulary for the target audience tends to enhance information processing fluency, making it easier for consumers to understand and take in brand-related information (27). This smoothness of processing, in turn, can positively influence consumers' cognitive, affective, and behavioral responses to the brand.

Research has found that when the content of an advertising information against consumers' deep-rooted stereotypes or biases, psychological resistance may arise, reducing acceptance and overall processing fluency (28). However, the language style employed to convey such information can play a crucial role. For instance, a message challenging a stereotype (e.g., novel informational content) might be processed with greater fluency if presented in a simple, direct, and unambiguous language style, as opposed to a complex or jargon-laden style. When the manner of expression (e.g., language style) facilitates comprehension and subtly navigates potential biases—perhaps through empathetic phrasing or a straightforward narrative structure, depending on the core factual claims—it can ease the cognitive burden on consumers, thus increasing their willingness to engage with and process the information. Therefore, advertising strategy should consider how stylistic choices interact with information content. By adopting clear and concise language, employing familiar syntactic patterns, and ensuring visual elements (e.g., typography) enhance readability, advertisements can improve information processing fluency. This stylistic facilitation can then promote positive brand perceptions and purchase intentions, even the information content itself is novel or requires a shift in perspective. For example, when introducing innovative nutrition products that might challenge existing consumer habits (e.g., informational novelty), employing a language style that emphasizes simplicity in its structure and uses accessible vocabulary to explain the product's easy use, compatibility, and positive impact can enhance consumers' information processing fluency. When consumers perceive that the provided information is easy to grasp, they are more likely to overcome resistance to the novelty of the information content and actively accept and try the new product.

Although numerous studies have explored the impact of advertising language on consumer behavior, a more nuanced understanding of how stylistic features (distinct from information content) influence information processing fluency as a mediator is still needed, especially when comparing different overarching language styles (e.g., disruptive vs. sustaining). Consumers' psychological motivations and cognitive processes significantly interact with the stylistic presentation of advertising information. For instance, a “disruptive” advertising style might be characterized by unconventional vocabulary, unexpected syntactic arrangements, or a bold tone, while a “sustaining” style might use more conventional, reassuring language and familiar sentence structures. If such a stylistic approach (disruptive or sustaining) aligns with consumers' self-identity or expectations for that product category, it can enhance their processing fluency for the information conveyed within that style, improving brand perception and goodwill. Therefore, advertising strategies should focus on crafting stylistic elements—both linguistic and visual—and an overall manner of information composition that resonates with target consumers' psychological expectations and cognitive processing capabilities (29). Such stylistically optimized strategies not only help consumers understand and accept advertising information more quickly but can also stimulate positive emotional responses and purchase intentions, by making the cognitive engagement easy and positive. Therefore, the following hypothesis has been proposed:

H2: Information processing fluency mediates the impact of advertising language style on consumer behavior.

### 2.3 The moderating role of information credibility

Information credibility refers to how much the source of information is trusted (30, 31). The important moderating role of information credibility cannot be ignored, in exploring how the advertising language style of nutrition products affects consumers' purchase intentions. When consumers are confronted with advertisements for nutrition products, they not only pay attention to the advertising language style (disruptive vs. sustaining), but also evaluate the authenticity and authority of the information. When the content of advertisements is based on scientific research, expert recommendations or certification by authoritative institutions, which means there is high information credibility of advertisements for nutrition products, both disruptive and sustaining advertising language styles are likely to enhance consumers' sense of trust, which in turn will increase their purchase intentions (32). While low information credibility has a greater impact on consumers' acceptance of advertising content, the differentiated effect of advertising language styles could be more significant at this time. Disruptive advertising language style attracts consumers' attention with its novelty and direct approach, which could quickly build up brand awareness and stimulate their purchase interest with high information credibility; however, over-exaggerated or unsupported expressions may trigger consumers' doubts with low information credibility. In contrast, sustaining advertising language style conveys information about the product in a gentle, step-by-step guided manner, which is more likely to deepen consumers' understanding and trust of the brand with high information credibility; with low information credibility, its steady style could reduce consumers' negative feelings, but it is equally difficult to stimulate their strong purchase intentions.

Thus, as a key factor, the degree of information credibility directly affects consumers' acceptance and trust in advertising information of nutrition product. With high information credibility, both disruptive and sustaining advertising language styles can more effectively convey the product value and enhance consumers' confidence in purchasing. Specifically, the novel and direct nature of disruptive advertising language style, can quickly attract consumers' attention and stimulate their desire to explore with high information credibility (33); whereas with low information credibility, it may cause consumers' doubts due to insufficient information or exaggerated expressions. In contrast, sustaining advertising language style deepens consumers' understanding and trust of the product with high information credibility, through gentle and step-by-step guidance; while with low information credibility, its robust style may alleviate consumers' mistrust to a certain extent, but the stimulation to purchase motivation could be relatively weak. Therefore, the degree of information credibility not only directly affects consumers' acceptance of adverting for nutrition product, but also regulates consumers' purchase intentions by interacting with different advertising language styles (34).

Based on the logic above, we further hypothesized that information credibility plays a moderating role in the impact of advertising language styles for nutrition products on consumers' purchase intentions, which varies between different advertising language styles (disruptive and sustaining). With higher information credibility, both disruptive and sustaining advertising language styles are more effective in conveying the symbolic values of nutrition products (e.g., health, vitality), which enhances consumers' purchase motivation and intentions. At this point, consumers are more inclined to believe the words in the advertisement and regard nutrition products as an important tool, to improve their self-image and quality of life. However, with lower information credibility, the disruptive advertising language style may trigger consumers' mistrust, due to its overly straightforward or exaggerated expression, thus weakening the effect of its symbolic value; while the sustaining advertising language style could alleviate this mistrust to a certain extent due to its gentle and steady style, but it is also difficult to fully stimulate consumers' purchase intentions. Therefore, with low credibility of information, the sustaining advertising language style may be more capable of maintaining consumers' purchase intentions. Therefore, the following hypothesis has been proposed.

H3: Information credibility (high vs. low) moderates the relationship between advertising language style (disruptive vs. sustaining) and consumers' purchase intentions.

**Figure 1:**
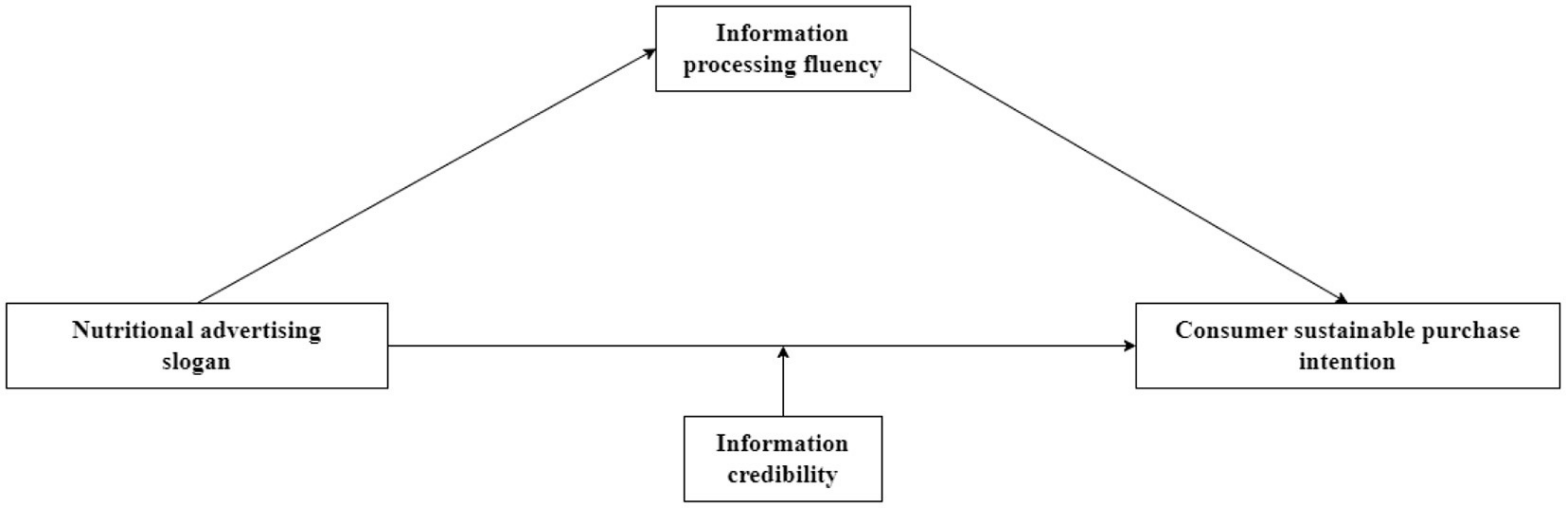
The conceptual model.

The conceptual model is shown in Figure 1.

## 3 Overview of the study

To verify the above three research hypotheses, a total of three related experiments were conducted. Experiment 1 examined the main effect of advertising language style (disruptive vs. sustaining) for nutrition products on consumers' purchase intentions, H1 was verified; Experiment 2 analyzed the mediating role of information processing fluency on the relationship between advertising language style for nutrition products and consumers' purchase intentions, H2 was verified; and Experiment 3 explored the moderating role of information credibility (high vs. low) on the relationship between advertising language style (disruptive vs. sustaining) for nutrition products and consumers' purchase intentions, H3 was verified. To better manipulate the language styles, different innovative advertising for nutrition products and scenario materials were designed for each experiment.

To ensure the successful manipulation of advertising language style, a manipulation check was conducted in each of these experiments (Experiments 1, 2, and 3). Specifically, after participants were exposed to either the disruptive or sustaining advertisement, they were asked to rate the perceived characteristics of the advertisement on several items with 7-point Likert-scale. These items were designed to assess perceptions of disruptiveness (e.g., “This ad is unconventional,” “This ad is novel,” and “This ad is groundbreaking”) and sustain-ness (e.g., “This ad is conventional” “This ad is traditional,” and “This ad is familiar”). The results of these manipulation check consistently confirmed our intended manipulation: participants in the disruptive advertising scenario rated the ads as significantly more disruptive and less sustaining compared to those in the sustaining advertising scenario (detailed statistical results for each experiment's manipulation check are provided within their respective methodology sections). This confirmed that our manipulation of advertising language styles was perceived by participants as intended. The frameworks associated with these three experiments are shown in Table 1. The pre-test results of the three experiments are shown in Table 2.

**Table 1:** The frameworks associated with these four experiments.

**Experiment**	**Experiment 1**	**Experiment 2**	**Experiment 3**
Purpose	To test for main effects (H1)	To test the mediating effect of information processing fluency (H2)	To test the moderating effect of information credibility (H3)
Independent variable	Advertising language style for nutrition products	Advertising language style for nutrition products	Advertising language style for nutrition products
Dependent variable	Consumers' sustainable purchase intention	Consumers' sustainable purchase intention	Consumers' sustainable purchase intention
Mediators	–	Information processing fluency	–
Moderator	–	–	Information credibility
Methods	ANOVA	ANOVA PROCESS 4	ANOVA PROCESS 1
Results	Supported H1	Supported H2	Supported H3

**Table 2:** The frameworks associated with these four experiments.

**Type of Experiment**	**Sample size**	***M***	**SD**	***F*-value**	***P*-value**
Experiment 1	205				
Experimental Group		5.13	1.48	25.08	< 0.001
Control group		4.33	0.60		
Experiment 2	200				
Experimental group		5.30	1.46	41.74	< 0.001
Control group		4.28	0.62		
Experiment 3	200				
Experimental group		4.33	0.63	29.99	< 0.001
Control group		1.48	0.15		

Participant Recruitment Criteria: Recruitment for our three experiments primarily adhered to the following inclusion criteria: (a) participants were adult residents of mainland China, aged 18 years or older; (b) they possessed the ability to fluently read and understand the Chinese questionnaires and experimental materials; and (C) they provided informed consent to participate in this study. Concurrently, we also implemented exclusion criteria, such as the removal of responses from participants who did not complete primary items, exhibited abnormal response times, or failed attention check questions. Efforts were also made to prevent duplicate participation. It must be acknowledged that despite our best efforts to use random sampling to control for sample bias, there may still be certain limitations in this study's sample. Participants who have completed the questionnaire and have had their data accepted, received a compensation of two Chinese yuan (~$0.28 USD).

## 4 Experiment 1: innovative advertising language style for nutrition products–consumers' purchase intentions

### 4.1 Experimental design

Experiment 1 aimed to investigate the main effect of advertising language style (disruptive vs. sustaining) for nutrition products on consumers' purchase intentions. In this study, G^*^power software was used to calculate the minimum sample size. We set the effect size to 0.25, α to 0.05, Power to 0.9, and the minimum sample size to 172. A one-way between-group experiment (advertising language style for nutrition products: disruptive vs. sustaining) was designed and 301 participants were recruited on Credamo (https://www.credamo.com/). Among the participants there were 151 (50.2%) males and 150 (49.8%) females. The age distribution of all the participants was 27.9% aged 18–25 years, 28.2% aged 26–40 years, 20.3% aged 41–60 years, and 23.6% aged 61 years and above. All participants were randomly divided into two groups, with 153 in the disruptive group; and 148 in the sustaining group.

**Figure 2:**
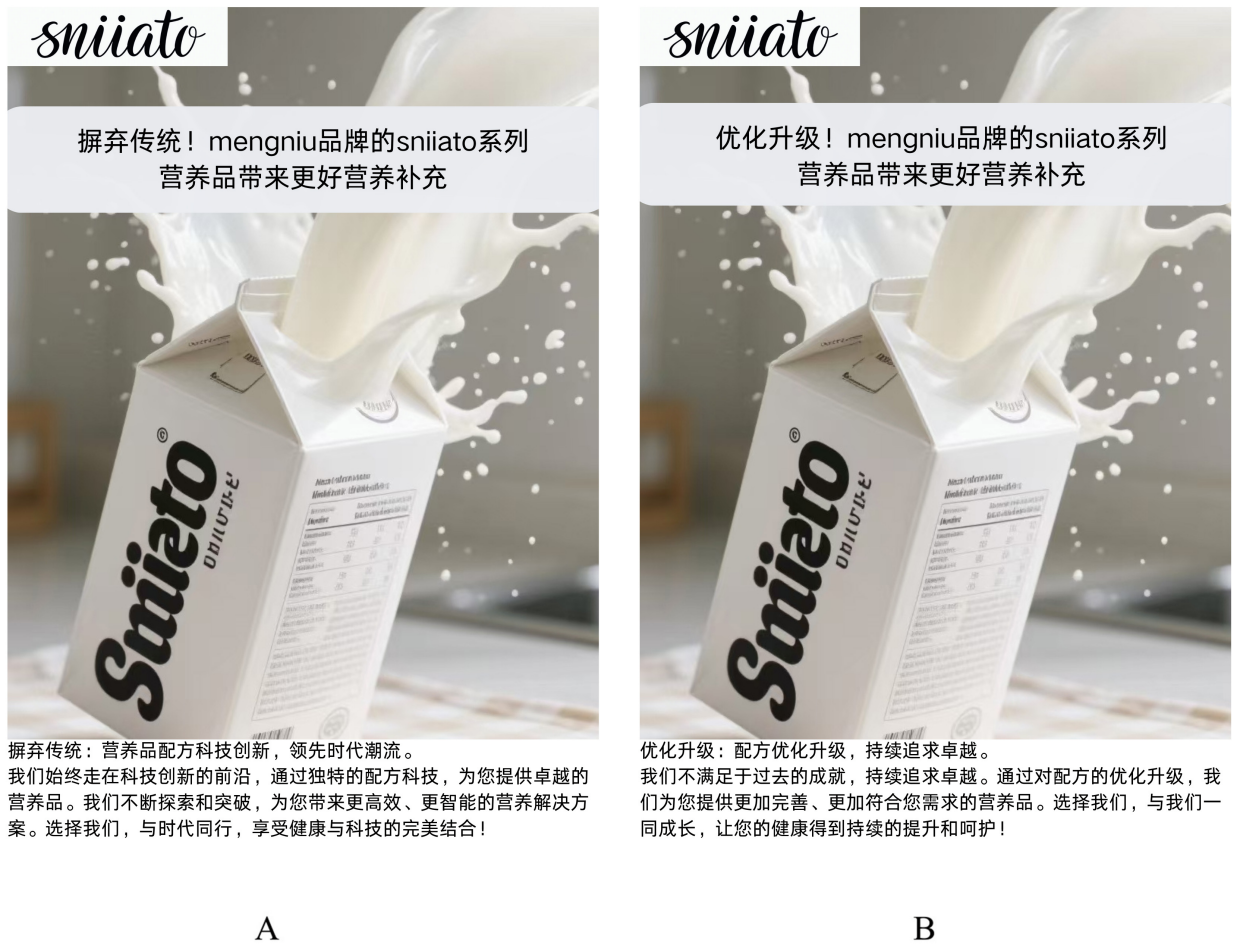
The advertising language for nutrition products in Experiment 1. **(A)** The stimulus material of the disruptive group; **(B)** Materials for sustaining the group.

Here is the experimental procedure. First, different scenario materials were used to guide the participants in two groups, to imagine that they need to buy nutrition products recently; the advertising language presented to the participants in the disruptive group was “Remove the dross, Mengniu nutrition products bring better nutritional supplements” and the advertising picture explained in detail, how the dross of tradition has been removed in certain products; while the advertising language presented to the participants in the sustaining group was “With optimization and upgrading, Mengniu nutritional products bring better nutritional supplements”, and the advertising picture elaborated how the nutrition product was optimized and upgraded. Then, participants were guided to answer the question “Do you agree that you would be willing to purchase the above nutrition products after learning about them?” (1 = Strongly Disagree, 7 = Strongly Agree) to measure the participants' purchase intention. Figure 2 shows the advertising style of nutrition products used in Experiment 1.

Given the study of Shamdasani et al. (35), consumers' brand attitude is an important influencing factor on consumers' purchase intention, which needs to be controlled. Participants were asked to answer two measurement questions about consumers' brand attitudes, such as “Do you agree that the Mengniu Dairy does not appeal to you?” (1 = strongly disagree, 7 = strongly agree) (35). Last, we collected demographic information related to participants.

### 4.2 Experimental results

Here is the main effect test. A one-way ANOVA was conducted with advertising language style (disruptive vs. sustaining) for nutrition products as the independent variable and consumers' purchase intentions as the dependent variable. The experimental results showed that the purchase intentions of the participants in the disruptive group (*M* = 6.33, SD = 1.323) was significantly higher than that of the sustaining group (*M* = 4.95, SD = 1.231), and *F*_(1, 299)_ = 87.74, *P* < 0.001. It can be seen that compared to products with the sustaining advertising language style, consumers are more inclined to purchase those with the disruptive advertising language style, which validated H1.

Given the study of Shamdasani et al. (35), which found that brand attitude has a significant effect on consumers' purchase intentions, brand attitude and gender were included as covariates in the Analysis of Covariance (ANCOVA) conducted in the present study. The experimental results indicate that, in this study, brand attitude and gender did not have a significant effect on consumers' purchase intentions [*F*_(2, 298)_ = 48.444, *P* < 0.001].

### 4.3 Discussion

Experiment 1 verified that disruptive advertising language style for nutrition products has a positive significant effect on consumers' purchase intentions, validating H1. Meanwhile, Experiment 1 illustrated brand attitudes do not have an effect on the experimental results, which enhances the accuracy of the findings. Despite the above findings in Experiment 1, the internal mechanism by which the advertising language style (disruptive vs. sustaining) for nutrition products affects consumers' purchase intentions has not been further explored. Therefore, Experiment 2 introduced information processing fluency as a mediating variable, in an attempt to verify its mediating role on the relationship between advertising language style (disruptive vs. sustaining) for nutrition products and consumers' purchase intentions.

## 5 Experiment 2: mediating role of information processing fluency

### 5.1 Experimental design

Experiment 2 was intended to investigate the effect of information processing fluency on consumers' purchase intentions. In this study, G^*^power software was used to calculate the minimum sample size. We set the effect size to 0.15, α to 0.05, Power to 0.95, and the minimum sample size to 214. We conducted a one-way between-group ANOVA (advertising language styles for nutrition products: disruptive vs. sustaining) and recruited 280 participants on Credamo (https://www.credamo.com/). A total of 267 valid questionnaires were collected. Ten participants who failed the attention test and three participants who filled in different questions with the same answers have been excluded. Among the participants, there were 132 (49.4%) male and 135 (50.6%) female. The age distribution of the participants was 26.2% aged 18–25 years, 30% aged 26–40 years, 20.6% aged 41–60 years, and 23.2% aged 61 years and above. All participants were randomly divided into two groups; there were 139 participants in the disruptive group; and 128 participants in the sustaining group.

**Figure 3:**
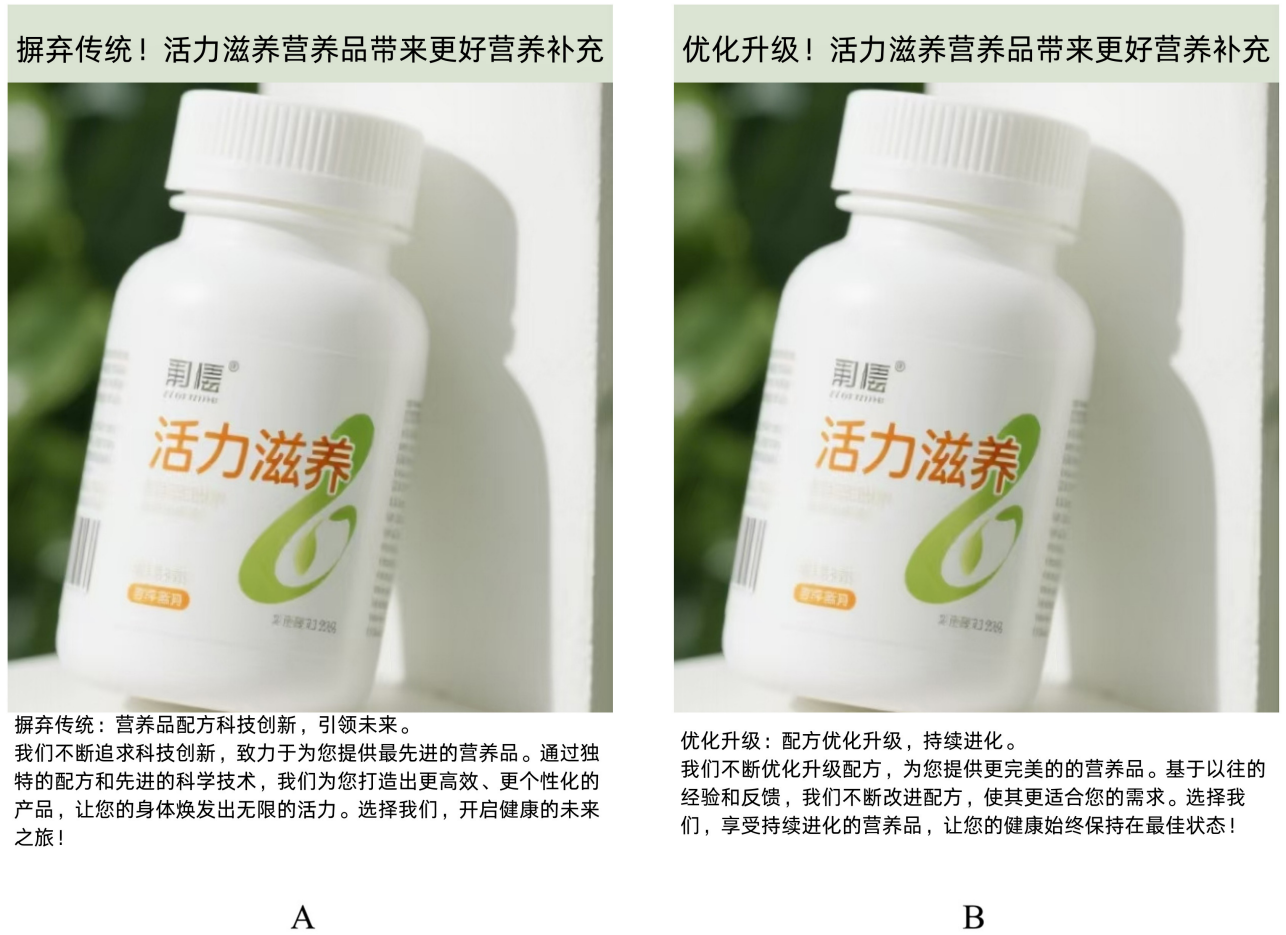
The advertising language for nutrition products in Experiment 2. **(A)** The stimulus material of the disruptive group; **(B)** Stimulus material for sustaining group.

Here is the experimental procedure. First, the same scenario material was used to guide the two groups of participants to imagine that they were shopping for a pharmaceutical product, while seeing an advertisement for the drug in a large pharmacy around their company; the same manipulation in Experiment 1 was employed again, with the replaced drug brand and stimulus material. Second, participants were asked 2 questions about information processing fluency, “Do you agree that the information conveyed with the advertising language style was easy to understand?” (1 = strongly disagree, 7 = strongly agree) (36). Along with this, the participants also answered questions about their purchase intentions. Last, we collected demographic information related to participants. The advertising language style for nutrition products used in Experiment 2 has been showed in Figure 3.

### 5.2 Experimental results

Here is the main effect test. One-way ANOVA was conducted with advertising language style (disruptive vs. sustaining) for nutrition products as independent variable and consumers' purchase intentions as dependent variable. The results of the experiment showed that consumers' purchase intentions (*M*_disruptive_ = 6.4, SD_disruptive_ = 1.214; *M*_sustaining_ = 4.88, SD_sustaining_ = 1.106), *F*_(1, 265)_ = 113.756, *P* < 0.001. It can be seen that consumers are more inclined to purchase nutrition products advertised in disruptive language style compared to those in sustaining language style. H1 was verified.

**Figure 4:**
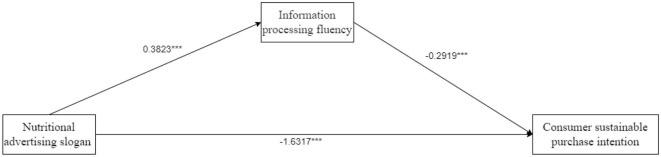
The mediating effect of information processing fluency in Experiment 2. ^*^p < 0.05; ^**^p < 0.01; ^***^p < 0.001.

Here is the mediating effect test. Taking advertising language style (disruptive vs. sustaining) for nutrition products as the independent variable, consumers' purchase intention as the dependent variable, and information processing fluency as the mediating variable, Process Model 4 was employed to verify the mediating role of information processing fluency (Bootstrap sample: 5,000) (37). The experimental results showed that the advertising language style for nutrition products had a significant effect on information processing fluency (β = 0.3823, *P* < 0.001); information processing fluency had a significant effect on consumers' sustainable purchasing (β = −0.2919, *P* < 0.001); and advertising language style for nutrition products had a significant effect on consumers' purchase intentions (β = −1.6317, *P* < 0.001). Overall, the mediation of advertising language style for nutrition products—information processing fluency—consumers' purchase intention was significant [β = 0.1116, SE = 0.0541, 95% CI = (0.0244–0.2329)]. It can be seen that information processing fluency fully mediates the relationship between advertising language style for nutrition products and consumer purchase intentions. H2 was verified. See Figure 4 for details.

Given the study of Sreen et al. (38), gender of the consumer is an important potential motivator that influences purchase intention. Therefore, a one-way ANOVA was conducted with gender as the independent variable and consumers' purchase intentions as the dependent variable. The results showed that gender had no significant effect on consumers' purchase intentions [*F*_(1, 265)_ = 0, *P* = 0.999].

### 5.3 Discussion

Experiment 2 verified the mediating role of information processing fluency on the relationship between the advertising language style for nutrition products and consumers' purchase intentions. Information processing fluency affects consumers' perception of and response to the advertising content, as well as the cost of information processing, thus affecting consumers' motivation and their purchase intentions. Meanwhile, it was found that the gender of consumers had no significant effect on the results of the experiment. Despite the above findings in Experiment 2, it has not been further explored whether there is a moderating effect between advertising language style for nutrition products and consumers' purchase intentions. Therefore, Experiment 3 introduced information credibility as a moderating variable to try to verify the moderating effect of information credibility on the relationship between advertising language style for nutrition products and consumers' purchase intentions.

## 6 Experiment 3: moderating role of information credibility

### 6.1 Experimental design

Experiment 3 was intended to verify the moderating role of information credibility on the relationship between advertising language style for nutrition products and consumers' purchase intentions. In this study, G^*^power software was used to calculate the minimum sample size. We set the effect size to 0.15, α to 0.05, Power to 0.95, and the minimum sample size to 214. We conducted a 2 (advertising language style for nutrition products: disruptive vs. sustaining) × 2 (information credibility: high vs. low) ANOVA and recruited 290 participants on Credamo (https://www.credamo.com/). Two participants who failed the attention test and four participants who filled out the questions with the same answers were excluded, collecting 284 valid questionnaires. Among all the participants, 138 (48.6%) were male and 146 (51.4%) were female. The age distribution was 26.4% aged 18–25 years, 30.3% aged 26–40 years, 20.4% aged 41–60 years and 22.9% aged 61 years and above. All participants were randomly divided into two groups, there were 142 participants in the disruptive group; and there were 142 participants in the sustaining group.

**Figure 5:**
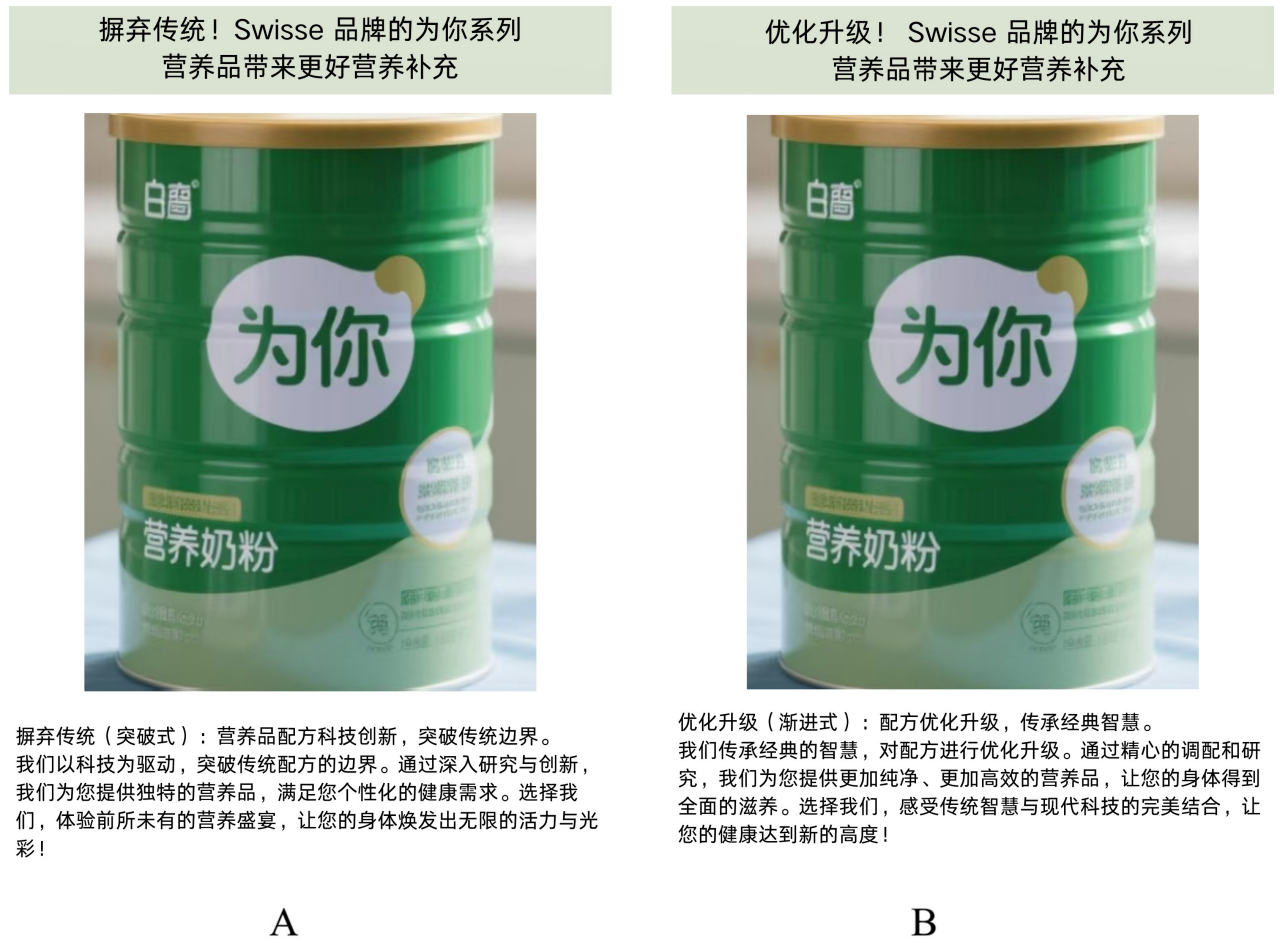
The advertising language for nutrition products in Experiment 3. **(A)** The stimulus material of the disruptive group; **(B)** Stimulus material for sustaining group.

Here is the experimental procedure. First, the same scenario material was used to guide the participants in both groups, to imagine that they were looking for a whey protein beverage on an international product sales website. The same manipulation method was employed as in Experiment 1, with international pharmaceutical brand Swisse and other different stimulus materials. Next, participants were asked three questions about the information credibility, such as, “Do you agree that you found that they were convincing?” (1 = Strongly Disagree, 7 = Strongly Agree), measurement questions were adapted from the study of Prendergast et al. (39). Then, participants answered measurement questions about purchase intention. Figure 5 shows the advertising style of nutrition products used in Experiment 3.

Advertising information is strongly subjective, and consumers' attitudes toward information are often an important influencing factor on consumers' purchase intentions. Therefore, consumers' attitudes toward information have been controlled, to enhance the robustness of the experimental results. Participants were asked two measurement questions about information attitudes, “Do you agree that, you will read the advertising information on the packaging when you buy nutrition products?” (1 = strongly disagree, 7 = strongly agree) (40). Last, we collected demographic information related to participants.

### 6.2 Experimental results

Here is the main effect test. A one-way ANOVA was conducted with advertising language style (disruptive vs. sustaining) for nutrition products as the independent variable and consumers' purchase intentions as the dependent variable. The experimental results showed that the purchase intention of the participants in the disruptive group (*M* = 6.58, SD = 0.599) was significantly higher than that in the sustaining group (*M* = 6.13, SD = 1.225), and *F*_(1, 282)_ = 15.502, *P* < 0.001. It can be seen that the advertising language style for nutrition products has a significant effect on consumers' purchase intentions, which verified H1.

**Figure 6:**
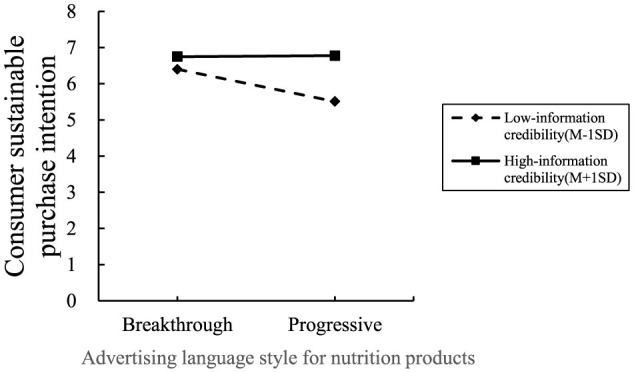
Experiment 3: result of interaction effect test.

Here is the moderating effect test. With advertising language style (disruptive vs. sustaining) for nutrition products as the independent variable, consumers' purchase intentions as the dependent variable, and information credibility as the moderating variable, Process Model 1 was employed to verify the moderating effect of information credibility on the relationship between advertising language style for nutrition products and consumers' purchase intentions (Bootstrap sample: 5,000) (37). The experimental results showed that: the path coefficient of advertising language style for nutrition products-consumers' purchase intentions is −0.4331^***^; the path coefficient of information credibility-consumers' purchase intention is 0.3953^***^; the interaction effect of advertising language style nutrition products and information credibility on consumers' purchase intention was significant [β = 0.4484, *P* < 0.001, 95% CI = (0.2527–0.6442)]. H3 was verified. See Figure 6 for details.

Given the findings of Koubaa et al. (41), which suggest that consumers' information attitude is an important factor influencing their purchase intentions, an Analysis of Covariance (ANCOVA) was conducted in the present study. Information attitude, gender, and age were included as covariates. The experimental results indicated that consumers' information attitude, gender, and age had no significant effect on the experimental results [*F*_(2, 281)_ = 26.637, *P* < 0.001].

### 6.3 Discussion

Experiment 3 verified the moderating effect of information credibility on the relationship between advertising language style (disruptive vs. sustaining) for nutrition products and consumers' purchase intentions. Specifically, with higher information credibility, consumers are more likely to be attracted by the strong visual effects and distinctive language of disruptive advertising language style, thus generating purchase intentions; with higher information credibility, sustaining advertising language style is able to gradually build up consumers' sense of trust and brand loyalty, so as to enhance their consumer purchase intentions through detailed information delivery. At the same time, the impact of consumers' information attitude on their purchase intention has been excluded.

## 7 General discussion

This study offers a new perspective on how advertising language styles (disruptive vs. sustaining) for nutrition products influence consumers' purchase intentions within the context of advertising innovation. The findings confirm that disruptive language in advertising innovation more effectively stimulates purchase intentions (H1), an effect partially mediated by information processing fluency (H2), and moderated by information credibility (H3). These findings contribute to both advertising theory and marketing practice, particularly concerning language strategies for promoting innovative nutrition products.

### 7.1 Theoretical implications

This study deepens the understanding of advertising language effects in several key aspects. First, it deepens the understanding of the impact of disruptive vs. sustaining styles. While prior research acknowledges the general influence of advertising language, this study offers a more nuanced understanding by directly comparing disruptive and sustaining styles in innovative nutrition products advertising. We found that disruptive language, with virtue of its novelty, is more adept at attracting initial attention and stimulating curiosity, which can turn into stronger purchase intentions. This finding extends existing literature by highlighting a specific mechanism through which disruptive styles operate, particularly for products defined as innovative. It is important not merely to restate that sustaining styles are less effective, but rather to explain why and under what conditions they might still be effective, or how they differ in processing. For instance, while sustaining styles might initially attract less attention to innovative products, their strength may lie in building trust or reinforcing existing positive attitudes over time, especially when innovation is incremental rather than disruptive.

Second, it elucidates the mediating role of information processing fluency. A key contribution is the identification of information processing fluency as a crucial mediator. This finding provides a cognitive-level explanation for how language styles turn into purchase intentions. Paradoxically, well-designed disruptive advertisements can enhance processing fluency (42), by making novel information surprisingly easy to understand, thereby leading to positive emotions and stronger purchase intentions. It challenges the simplistic assumption that all “disruptive” stimuli are inherently difficult to process. For sustaining styles, fluency may be achieved through familiarity and clarity; however, this may be insufficient to drive purchase intentions for innovative products without an additional spark. This not only underscores the importance of the information itself, but also of how fluently consumers can process it, which is directly linked language style to cognitive ease.

Finally, it highlights the moderating role of information credibility: the moderating effect of information credibility provides a critical boundary condition. Our results emphasize that even the most creative or fluent advertising information will be significantly less effective if their source or content is perceived as untrustworthy. High credibility acts as an amplifier, enabling both disruptive and sustaining styles to persuade more effectively. Conversely, low credibility significantly diminishes positive effects, indicating that establishing foundational trust is paramount before sophisticated language strategies can yield optimal returns (43). This finding integrates source credibility research with the literature on advertising styles, emphasizing their interactive influence on consumer persuasion, particularly in a market like nutrition products where credibility is often a key consideration.

### 7.2 Practical implications

The findings of this study offer several actionable insights for marketers of nutrition products: first, the strategic selection of language style: marketers should strategically choose between disruptive and sustaining language styles based on specific marketing objectives and the perceived innovativeness of the product. For launching genuinely novel nutrition products or variants, a disruptive style can be a powerful tool to break through market clutter and generate initial interest. However, this must be balanced with ensuring the information is clear and credible. For more established products or incremental innovations, a sustaining style may be more suitable for reinforcing brand value and building long-term trust, provided it is sufficiently engaging.

Second, emphasizing the optimization of information processing fluency in advertising. Regardless of the chosen style, marketers must prioritize information processing fluency. For disruptive advertisements, this means ensuring their novelty is clever and easily understandable, not merely confusing. Visual cues, clear calls-to-action, and intuitive navigation (if digital) can help enhance fluency. For sustaining advertisements, clarity, logical flow, and easily understandable language are key. Avoiding jargon and presenting information in an easily digestible format will enhance fluency. Prioritizing the establishment and communication of information credibility: given the strong moderating role of credibility, establishing and communicating trust is indispensable. This can be achieved through transparent ingredient sourcing, third-party certifications and endorsements (e.g., nutritionist approvals, scientific backing), and authentic user reviews and recommendations.

Finally, informing broader communication strategies: beyond traditional advertising, our findings can inform several other communication touchpoints. Food labeling: for nutrition products, especially those with innovative formulations, packaging and labels can utilize a mildly disruptive yet clear language style to highlight unique benefits (e.g., using “Next-Gen Gut Health” instead of just “Probiotics”). However, core nutritional information must be maintained in a highly fluent and credible (sustaining) format to comply with regulations and build consumer trust. App-based “nudges”: health and nutrition apps can apply these principles to in-app messages or “nudges.” For example, a disruptive notification (Shake up your snack routine with our new protein bar!) might encourage trial of a new feature or product, while a sustaining message (Remember your daily hydration goal!) reinforces existing healthy habits. The perceived credibility of the app itself will moderate the effectiveness of these nudges. Social media strategies: on social media, where attention is scarce, disruptive content can increase engagement and viral spread. However, this should be balanced with consistent, credible information about product benefits and a clear brand voice. When collaborating with influencers, priority should be given to those with high perceived credibility to amplify the information, regardless of whether the campaign's core information is disruptive or sustaining. The key is to align the language style with platform norms and audience expectations for credibility and fluency.

### 7.3 Limitations and future research directions

It is necessary to acknowledge the limitations of this study, in terms of sample selection. First, the sample was mainly based on a selected group and may not have comprehensively covered consumers from different ages, genders, cultural backgrounds and socio-economic statuses, thus limiting the generalizability and wide applicability of the study results. Future research should expand the sample to ensure the coverage of diverse consumer groups and conduct cross-cultural comparative studies, to enhance the generalizability and broad representativeness of the findings. Second, this study mainly focused on the effects of advertising language style on consumers' short-term purchase intentions, but did not address the changes in long-term purchase behavior. Therefore, the findings may not fully reflect the long-term effects of advertising language styles on consumers' sustainable purchase behavior. Future research could adopt a longitudinal research design to track and observe the changes in consumers' long-term purchasing behaviors, after exposure to different advertising language styles, so as to more accurately assess advertising language styles by collecting long-term data. In addition, this study primarily focuses on purchase intention rather than actual purchase behavior. Therefore, it employs subjective questionnaires for data collection, relying on participants' self-reports. For future research, it is recommended to focus on the following directions, such as eye-tracking experiments, electroencephalogram (EEG) experiments, or studies based on actual purchase behaviors.

## Data Availability

The original contributions presented in the study are included in the article/supplementary material, further inquiries can be directed to the corresponding authors.
